# Recurrence of Transverse Colon Cancer in Diverticulum 14 Years after Endoscopic Mucosal Resection: A Case Report

**DOI:** 10.70352/scrj.cr.25-0700

**Published:** 2026-04-17

**Authors:** Kazuki Shimomura, Hiromitsu Iwamoto, Kenji Matsuda, Yasuyuki Mitani, Yuki Nakamura, Norio Takemoto, Toshihiro Sakanaka, Masato Tamiya, Takahiko Hyo, Katsuya Ueda, Mizuki Nishikawa, Manabu Kawai

**Affiliations:** 1 Second Department of Surgery, School of Medicine, Wakayama Medical University, Wakayama, Wakayama, Japan; 2 Department of Human Pathology, School of Medicine, Wakayama Medical University, Wakayama, Wakayama, Japan

**Keywords:** colonic diverticulum, diverticulosis, colon cancer, endoscopic submucosal resection, diverticulum carcinoma, recurrence

## Abstract

**INTRODUCTION:**

Carcinoma arising from a colonic diverticulum is extremely rare, with only 21 cases being reported in the literature. Accurate pathological assessment of lesions within diverticula is often challenging due to distortion or discontinuity of the muscularis mucosae, which may lead to misjudgment of invasion depth and inappropriate therapeutic decisions.

**CASE PRESENTATION:**

We report the case of a 74-year-old female with a history of left breast cancer and prior endoscopic mucosal resection (EMR) for a 7-mm transverse colon cancer 14 years earlier. Histopathology at that time revealed a moderately differentiated adenocarcinoma with submucosal invasion of 800 μm, negative vertical and horizontal margins, and no indication for additional surgery. Surveillance colonoscopy at 6 months showed only a scar, but then she was lost to follow-up. After detection of an elevated carcinoembryonic antigen level of 56.8 ng/mL, the patient was referred to us. A lesion adjacent to the transverse colon was seen on CT and PET, but no abnormalities were seen on colonoscopy. We performed laparoscopic transverse colectomy with D2 lymph node dissection. Histopathological examination revealed a tumor centered in the subserosal/pericolic tissue, histologically identical to the prior EMR specimen, staged as pT3, and consistent with local recurrence of the original lesion. Re-evaluation of the original EMR slides with desmin immunostaining confirmed that the tumor had arisen within a diverticulum, and the true invasion depth was 4000 μm rather than the initially reported 800 μm. The patient recovered uneventfully, underwent laparoscopic incisional hernia repair 1 year later, and remains recurrence-free 24 months after colectomy.

**CONCLUSIONS:**

This case highlights the diagnostic difficulty of accurately assessing invasion depth in tumors arising within diverticula. Misinterpretation may result in underestimation of metastatic risk and inadequate treatment. In this case, recurrence of colon cancer was detected 14 years after EMR. Diverticula should always be carefully documented during colonoscopy and should be considered in pathological interpretation. Recurrence may occur many years after endoscopic therapy, so close communication between endoscopists and pathologists is essential, and long-term surveillance is warranted.

## Abbreviations

EMR
: endoscopic mucosal resection
NCCN
: National Comprehensive Cancer Network

## INTRODUCTION

Diverticulosis of the colon is common, with a particularly high prevalence in Western countries. The prevalence of diverticulosis increases with age, and it affects approximately 5% of individuals <40 years old, 50% of those in their 60s, and 71% of those ≥80 years old.^1)^ Most cases are asymptomatic and are detected incidentally during abdominal CT or colonoscopy. The exact etiology of diverticulum formation remains unclear, but potential risk factors include genetic predisposition; environmental factors such as low dietary fiber intake, high red meat consumption, alcohol intake, and smoking^2)^; and microenvironmental factors, including alterations in the intestinal microflora and low-grade inflammation.^3)^ Colorectal cancer and diverticulosis share certain risk factors, but a causal relationship has not been established.^4)^ Carcinoma arising directly from a colonic diverticulum is extremely rare, with only 21 cases reported in the literature to date.

Herein, we describe a case of transverse colon cancer that recurred 14 years after EMR, despite histopathological confirmation of negative vertical and horizontal margins at the initial procedure and the absence of an indication for additional surgical resection.

## CASE PRESENTATION

A 74-year-old female had a high carcinoembryonic antigen level. She was receiving medication for hypertension and osteoporosis from her family doctor, and the finding was noted during a routine outpatient blood test. Approximately 28 years previously, she had undergone surgery for left breast cancer, but there was no evidence of breast cancer recurrence at this time.

**Fig. 1:**
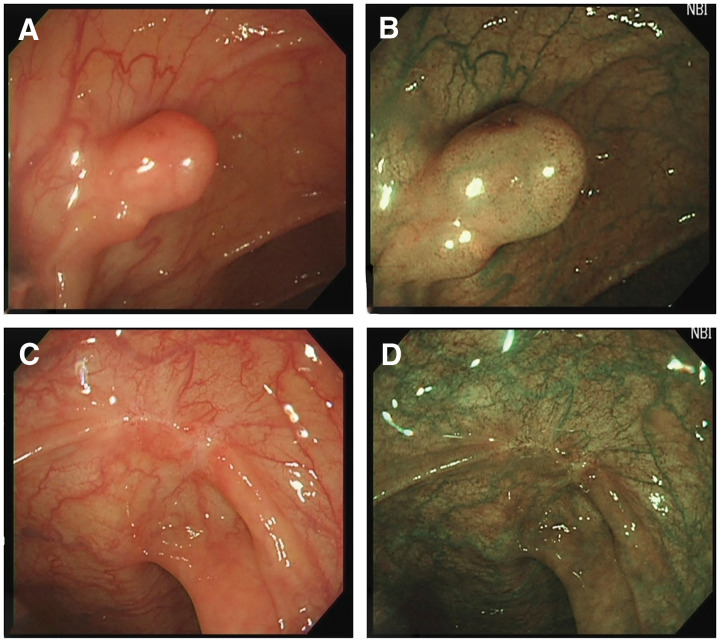
Colonoscopy at the time of EMR 14 years ago. A 7-mm-sized type 0–II lesion was found in the transverse colon by colonoscopy and was resected en bloc by EMR (**A**, **B**). Six months after EMR, only a scar was detected at colonoscopy (**C**, **D**). EMR, endoscopic mucosal resection

Approximately 14 years before the current presentation, the patient had undergone EMR for a 7-mm-sized early-stage transverse colon cancer (**Fig. 1**). The histopathology results at that time showed a moderately differentiated adenocarcinoma with pSM: 800 μm, med, IFNb, ly0, v0, pHM0, pVM0. She underwent a surveillance colonoscopy 6 months after treatment, leaving only a post-treatment scar and no residual tumor. The doctor at the hospital where the patient underwent EMR recommended annual surveillance, but this recommendation was not followed.

**Fig. 2:**
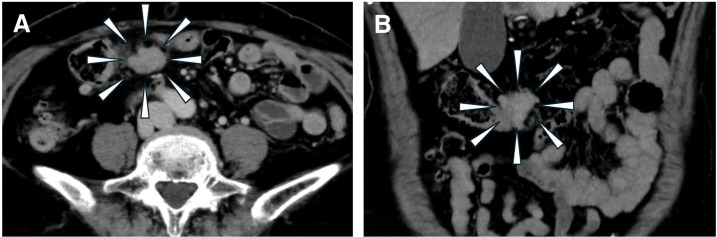
Contrast-enhanced CT of the abdomen. Axial (**A**) and coronal views (**B**). A 40-mm tumor borders the middle of the transverse colon, with a pale contrast effect.

**Fig. 3:**
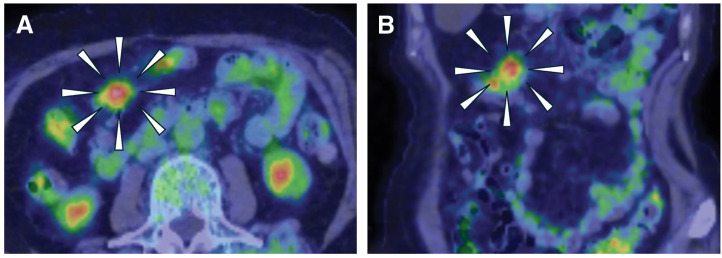
Positron emission tomography. Axial (**A**) and coronal views (**B**). An accumulation of SUVmax = 5.17 is shown in the tumor bordering the transverse colon. Other physiologic accumulations were observed. SUVmax, maximum standardized uptake value

**Fig. 4:**
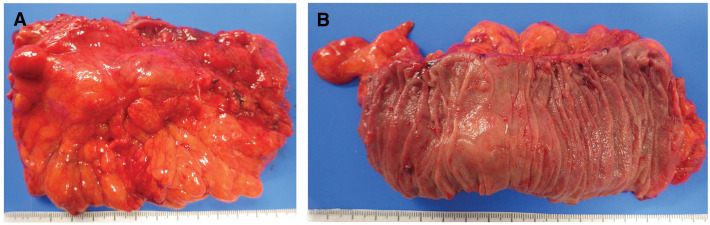
Specimen removed during surgery. The transverse colon mesentery and serosal surface showed traction and indentation (**A**), but no obvious tumor or EMR scar was observed on the mucosal surface (**B**).

**Fig. 5:**
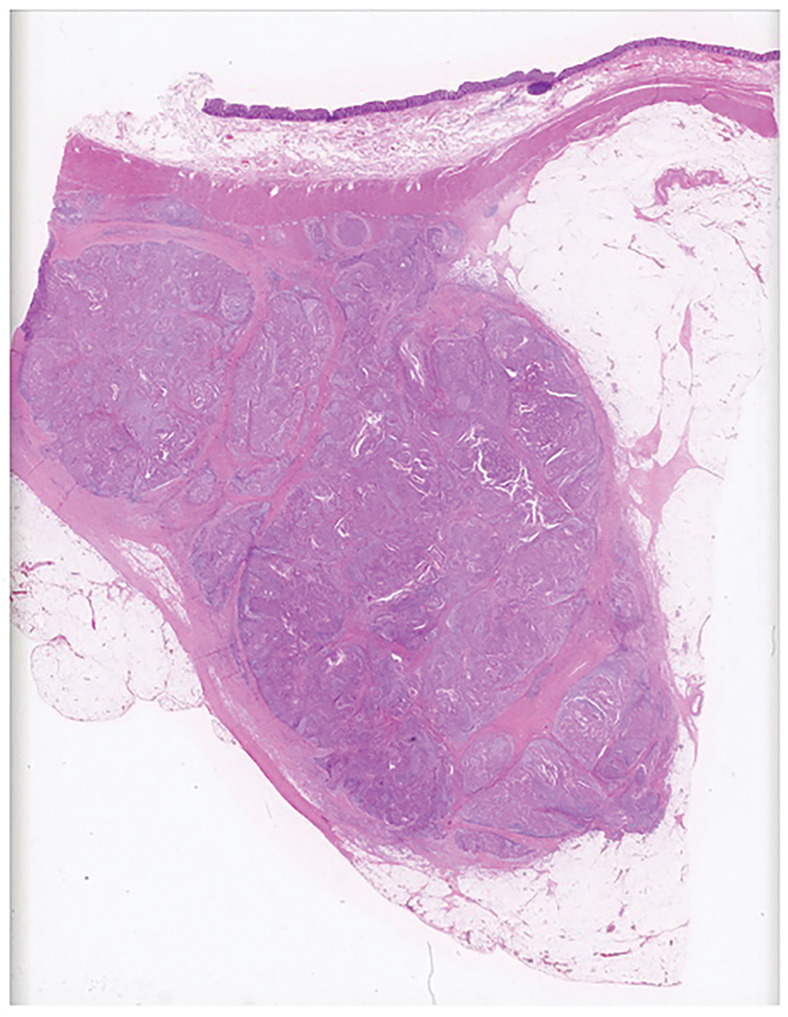
Pathology of the surgical specimen. HE stain shows tumor cell infiltration mainly in the subserosa, and the lesion was diagnosed as pT3. No tumor cell infiltration was identified in the mucosa or submucosa. HE, Hematoxylin–eosin

Fourteen years after the EMR, an elevated carcinoembryonic antigen tumor marker was detected in a blood test taken by her family doctor (56.8 ng/mL, normal range ≤5.0 ng/mL), which led to a visit to a nearby general hospital. CT showed an intra-abdominal tumor with a pale contrast effect on the side of the transverse colon (**Fig. 2A** and **2B**). Colonoscopy showed no obvious abnormal findings on the mucosal surface, including the EMR scar. The patient was referred to our department because PET also showed an accumulation with a maximum standardized uptake value (SUVmax) of 5.17 at the same site (**Fig. 3A** and **3B**). Contrast enema or CT colonography was not performed. Based on a comprehensive evaluation of the previous examination results and considering the possibility of transverse colon cancer recurrence, we performed a laparoscopic transverse colon resection and D2 lymph node dissection. Preoperative marking was not performed, but laparoscopic examination revealed traction to the transverse mesocolon consistent with the preoperative diagnosis, indicating the tumor location. Following standard transverse colon cancer resection techniques, we secured resection margins of 10 cm both proximally and distally (**Fig. 4A** and **4B**). Histopathological examination revealed a tumor centered in the subserosal/pericolic tissue, and no tumor cell infiltration was identified in the mucosa or submucosa (**Fig. 5**). It was histologically identical to the prior EMR specimen, staged as pT3, and no metastasis was detected in the dissected lymph nodes (pN0). The patient was discharged home 11 days after surgery without postoperative complications. Six months after surgery, she returned with abdominal distension, and an abdominal wall scar hernia was found in the midline wound. A further 6 months after that, we performed a laparoscopic ventral incisional hernia repair (intraperitoneal onlay mesh plasty plus, sometimes known as “IPOM Plus”). At the time of writing, the patient is currently undergoing outpatient surveillance without postoperative adjuvant chemotherapy. She has had no recurrence of either transverse colon cancer at 24 months postoperatively or abdominal wall scar at 12 months postoperatively.

## DISCUSSION

The pathological examination of the surgically resected specimen demonstrated a lesion centered in the subserosal/pericolic tissue, and its histology closely resembled that of the pT1 transverse colon cancer resected by EMR 14 years previously. The original EMR slides were reviewed again and demonstrated moderately differentiated tubular adenocarcinoma (tub2, pT1b [SM 4000 μm], Ly0 [D2–40], V0 [Vb-HE], Pn0, BD1, pVM0, pHM0). We attributed the discrepancy from the initial report of SM 800 μm to differences in the reference baseline for measuring submucosal invasion within a diverticulum, where the muscularis mucosae may be distorted, displaced, or focally discontinuous. Pathological criteria for pT1 colorectal cancer as an indication for additional resection after endoscopic treatment are described in the NCCN clinical practice guidelines in oncology for colon cancer^5)^ and are widely used in daily practice. A risk assessment method for lymph node metastasis of pT1 colorectal cancer after endoscopic treatment has recently been reported^6)^ and is gradually becoming popular. Although there have been reports of similar risks between colorectal diverticulum and colorectal cancer,^2,3)^ the direct causal relationship between colorectal diverticulosis and colorectal cancer remains unknown.^4)^

In our patient’s case, the depth recorded at the time of EMR was SM 800 μm. Using the nomogram reported by Kajiwara et al.,^7)^ the estimated risk of lymph node metastasis at that time was calculated as follows: gender, female (30 points); site, transverse colon (0 points); histology, moderately differentiated adenocarcinoma (37.5 points); lymphovascular invasion, negative (0 points); tumor budding, BD1 (0 points); depth, 800 μm (0 points). The total score of 67.5 corresponded to an approximate nodal risk of approximately 1%. If the depth had been assessed as SM 4000 μm (adding 100 points), the total would have been 167.5, with an estimated risk of about 4%. Although depth is the most influential variable in the model, neither estimate indicated a high probability of nodal metastasis.

**Fig. 6:**
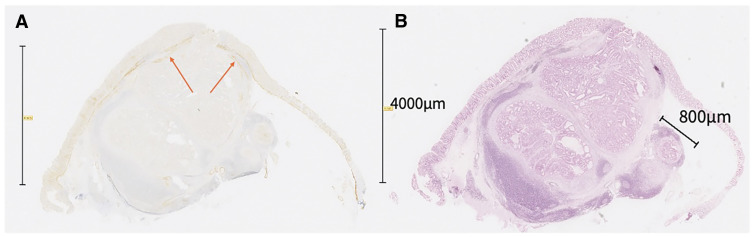
Pathology at the time of EMR 14 years previously. (**A**) Desmin stain: Desmin-positive muscularis mucosae stained in an arcuate pattern (arrow), suggesting a tumor within the diverticulum. (**B**) HE stain: The diverticulum is filled with tumor cells, suggesting diverticular carcinoma. The depth of the tumor was 800 μm when measured from the closest muscularis mucosae. The depth of the tumor was 4000 μm when measured from the most superficial muscularis mucosae.

To clarify the relationship between the current lesion and the prior EMR site, we re-evaluated the EMR specimen. Desmin immunostaining delineated the muscularis mucosae, and its configuration suggested that the tumor had arisen within a diverticulum. The previously reported depth of SM 800 μm appears to have been measured from the nearest muscularis mucosae plate at a diverticular fold (**Fig. 6A**). HE staining demonstrated invasion measured at 4000 μm from the muscularis mucosae (**Fig. 6B**).

Pathological assessment of the present surgical specimen showed a tumor centered in the subserosa/pericolic adipose tissue and staged as pT3. No tumor cell infiltration was identified in the mucosa or submucosa of the resected segment, and the histologic features mirrored those of the original EMR specimen. These findings support a diagnosis of local recurrence of the transverse colon cancer resected by EMR 14 years earlier, an extremely rare clinical course.

We infer that the discrepancy in depth assessment stemmed from the tumor’s origin within a colonic diverticulum. Colonic diverticula are classified as true or false (pseudo-diverticula). In pseudo-diverticula, there is no intrinsic muscular layer, which complicates depth measurement based on the muscularis mucosae. In endoscopically resected specimens, it is often difficult to determine whether a lesion arose from a true or false diverticulum, and particular caution is warranted when assessing depth in lesions arising in pseudo-diverticula.

A PubMed search using the keywords “colorectal cancer,” “adenocarcinoma,” “diverticular disease,” and “diverticulum” identified just 21 reported cases of colorectal cancer arising within a colonic diverticulum between 1967 and 2024 (**Table 1**). Prognosis and recurrence rates specific to diverticular carcinoma have not been systematically characterized, and long-term outcomes remain uncertain. Endoscopic therapy carries a relatively high risk of perforation in this setting.^8)^ Among the 21 reported cases, patients underwent endoscopic treatment in only 2 cases,^9,10)^ both of which were complicated by a perforation during endoscopic treatment. Currently, there are no established international guidelines for the management of the lesions arising from colonic diverticula. In Japan, guidelines for colonic diverticular disease^11)^ are available; however, they mainly focus on diverticulitis and diverticular bleeding and do not provide specific recommendations for endoscopic treatment of the lesion arising from colonic diverticula. There are no reports in the literature of additional resection after endoscopic treatment for colon cancer arising in a diverticulum. In our patient, the diverticular component was not recognized at the time of EMR, yet the procedure was completed without perforation, and surveillance colonoscopy at 7 months demonstrated only a post-treatment scar (**Fig. 3B**). Although rare, the possibility of diverticulum-associated carcinoma should be considered during diagnostic colonoscopy and when planning endoscopic therapy.

**Table 1:** Clinical characteristics of reported cases of colon cancer arising from a diverticulum

Case	Author	Year	Age (years)/sex	Symptom	Preoperative diagnosis	Tumor site	Treatment	True/pseudo-diverticulum	Differentiation	Tumor size (mm)	TNM (UICC)	Outcome (months)
1	Tolley^12)^	1967	59/M	Constipation	Diverticulitis	Cecum	Right hemicolectomy	ND	Mucinous	ND	T4N0M0	24, no recurrence
2	Beal^13)^	1971	65/F	Abdominal pain	Diverticulitis	Left	Left hemicolectomy	Pseudo	ND	ND	ND	Alive, duration unknown
3	Drut^14)^	1974	84/M	Abdominal pain	Diverticulitis	Sigmoid	Left hemicolectomy	ND	ND	60	T4N3M0	Died on POD 1
4	Hines and Gordon^15)^	1975	55/F	Abdominal pain	Diverticulitis	Descending	Left hemicolectomy	Pseudo	Mucinous	ND	T4N0M0	Died of other causes at 20 M
5	McCraw et al.^3)^	1976	80/M	Hematochezia	Diverticulitis	Sigmoid	Sigmoidectomy	ND	Mucinous	ND	ND	Died of other causes at 36 M
6	Prescott et al.^16)^	1992	89/F	Abdominal pain	Giant diverticulum	Sigmoid	Sigmoidectomy	Pseudo	Well	30	T3N0M0	ND
7	Cohn et al.^17)^	1993	80/M	Abdominal pain	Ileus	Sigmoid	Hartmann operation	Pseudo	Mucinous	17	T3N0M0	15, no recurrence
8	Cohn et al.^17)^	1993	61/M	Hematochezia	Bulky tumor	Sigmoid	Sigmoidectomy	True	Well	45	T2N0M0	3, no recurrence
9	Kajiwara et al.^18)^	1996	67/M	Hematochezia	Cancer	Ascending	Right hemicolectomy	Pseudo	Mucinous	15	T3N0M0	12, no recurrence
10	Kikuchi et al.^19)^	1999	58/F	No symptom	Tumor	Cecum	Ileocecal resection	Pseudo	Moderately	6	TisNxM0	Alive, duration unknown
11	Bellows et al.^20)^	2002	63/M	Hematochezia	Vesicosigmoidal fistula	Sigmoid	Sigmoidectomy	ND	Moderately	ND	T4aN0M0	ND
12	van Beurden, et al.^21)^	2008	54/M	Diverticultis	Diverticulitis	Cecum	Sigmoidectomy	ND	Moderately	20	T4aN2M0	12, no recurrence
13	Fu et al.^9)^	2010	71	ND	Tumor	Descending	EMR with the assistance of laparoscopy	Pseudo	Well	15	TisNxM0	ND
14	Soga et al.^22)^	2011	71/M	Hematochezia	Diverticular bleeding	Ascending	Right hemicolectomy	Pseudo	Well	20	ND	ND
15	Merkow and Sun^23)^	2011	60/M	Abdominal pain	Bulky tumor	Sigmoid	Sigmoidectomy	ND	Poorly	40	T4bN2M0	ND
16	Parsyan et al.^24)^	2013	60/M	ND	Cancer	Sigmoid	Left hemicolectomy	True	Moderately	70	T3N1M0	ND
17	Imai et al.^8)^	2014	81/M	No symptom	Cancer	Ascending	Right hemicolectomy	True	ND	25	T1bN0M0	ND
18	Yagi et al.^25)^	2014	73/M	Hematuria	Cancer	Sigmoid	Sigmoidectomy, total resection of the urinary bladder	ND	Well	ND	T4N0M0	ND
19	Yoshida et al.^10)^	2017	68/F	No symptom	Cancer	Ascending	EMR	ND	ND	30	T1bN0M0	ND
20	Kimura et al.^26)^	2018	55/M	Left inguinal swelling	Cancer	Sigmoid (2 lesions)	Laparoscopic sigmoidectomy	ND	Moderately	ND	ND	ND
21	Kayano et al.^27)^	2019	76/M	Dysuria	Vesicosigmoidal fistula	Sigmoid	Laparoscopic sigmoidectomy	ND	Well	ND	T4bN0M0	18, recurrence in bladder wall
22	Our case	2026	74/F	No symptom	Recurrence of cancer	Transverse	Laparoscopic transverse colectomy	Unknown	Moderately	25	T3N0M0	24, no recurrence

F, female; M, male; ND, not described; TNM, tumor, node and metastasis classification; UICC, Union for International Cancer Control

## CONCLUSIONS

The presence of diverticula should always be noted during colonoscopy and endoscopic treatment, as its effects may influence the pathological diagnosis. The possibility of previous recurrence should also be considered when there is suspicion of malignancy. This case suggests the importance of 2-way communication between clinicians and pathologists, with each sharing information obtained during treatment and diagnosis. Regular follow-up is important because of the possibility of recurrence even more than 10 years after endoscopic treatment.

## DECLARATIONS

### Availability of data and materials

The datasets used during the current study are available from the corresponding author upon reasonable request.
